# Trans-1,3-diphenyl-2,3-epoxypropan-1-one, a chalcone derivative, induces apoptosis via ROS-mediated down-regulation of Bcl-xL in human leukemia HL-60 cells

**DOI:** 10.17179/excli2015-373

**Published:** 2015-08-03

**Authors:** Eun-Yi Ko, Seung-Hong Lee, Ji-Yeon Ko, Jeong Yong Moon, Weon-Jong Yoon, Ginnae Ahn, Seong Woon Roh, Kichul Cho, You-Jin Jeon, Daekyung Kim, Kil-Nam Kim

**Affiliations:** 1 Jeju Center, Korea Basic Science Institute (KBSI), Jeju 690-140, Republic of Korea; 2 School of Marine Biomedical Sciences, Jeju National University, Jeju 690-756, Republic of Korea; 3 Division of Food Bioscience, Konkuk University, Chungju Chungbuk 380-701, Republic of Korea; 4 Subtropical Horticulture Research Institute, Jeju National University, Jeju 690-756, Republic of Korea; 5 Jeju Biodiversity Research Institute, Jeju Technopark, Jeju, 699-943, Republic of Korea; 6 Department of Marine Bio-Food Sciences, Chonnam National University, Yeosu 550-749, Republic of Korea; 7 Department of Marin Biotechnology, University of Science and Technology, Daejeon 305-350, Republic of Korea

**Keywords:** anticancer, trans-1,3-diphenyl-2,3-epoxypropan-1-one (DPEP), apoptosis, reactive oxygen species (ROS), Bcl-xL

## Abstract

The anticancer effects of trans-1,3-diphenyl-2,3-epoxypropan-1-one (DPEP), a chalcone derivative, were investigated in human leukemia HL-60 cells. Treatment of HL-60 cells with various concentration of DPEP resulted in a sequence of events characteristic of apoptosis, including loss of cell viability, morphological changes, and increased sub-G_1_ DNA content. We demonstrated that DPEP elevates reactive oxygen species (ROS) levels in HL-60 cells, and that the ROS scavenger N-acetylcysteine (NAC) could block DPEP-induced ROS generation and apoptosis. Western blot analysis revealed that DPEP inhibits Bcl-xL expression, leading to caspase-3 activation and poly-ADP-ribose polymerase (PARP) cleavage, thereby inducing apoptosis. However, NAC pre-treatment significantly inhibited the activation of caspase-3 and PARP cleavage and reduced Bcl-xL levels. These findings provide the first evidence that DPEP may inhibit the growth of HL-60 cells and induce apoptosis through a ROS-mediated Bcl-xL pathway.

## Introduction

Apoptosis is a fundamental cellular event during development and is critical for anticancer drug-induced cytotoxicity (Cotter, 2009[7]). Apoptosis can be triggered by numerous stimuli, including cellular reactive oxygen species (ROS), reactive nitrogen species (RNS), hormones, cell-cell interactions, growth factor withdrawal, antigens, and chemotherapeutic agents (Chen et al., 2011[5]; Ou et al., 2010[17]). ROS, most notably the superoxide anion radical (·O_2_^-^), hydrogen peroxide (H_2_O_2_), and the hydroxyl radical (·OH), are unwanted metabolic byproducts of normal aerobic metabolism. Basal levels of ROS serve as physiological regulators of normal cell proliferation and differentiation; however, excessive levels of ROS can inhibit tumorigenesis by inducing DNA damage- and oxidative stress-mediated apoptosis (Valko et al., 2006[22]; Trachootham et al., 2009[21]; Kim et al., 2010[12]). Furthermore, previous reports indicate that ROS levels are increased when cells are exposed to various compounds, including paclitaxel and other anticancer therapeutics (Alexandre et al., 2007[3]). These studies suggest that ROS-induced DNA damage and oxidative stress represent a major anti-cancer mechanism among chemopreventive agents (Rigas and Sun, 2008[18]). Thus, ROS plays an important role in anticancer drug-mediated apoptosis. 

In recent years, chalcones (1,3-diaryl-2-propen-1-ones) have received a great deal of attention due to their diverse biological functions. Chalcones constitute an important class of natural products belonging to the flavonoid family, and display anti-inflammatory, antibacterial, antioxidant, antimalarial, and anticancer activity (Cheng et al., 2008[6]; Kim et al., 2013[13]; Abdullah et al., 2014[1]; Tadigoppula et al., 2013[20]). Importantly, chalcone derivatives have also been shown to have anticancer activity (Na and Nam, 2011[16]; Zhang et al., 2011[26]). However, the anticancer activity of trans-1,3-diphenyl-2,3-epoxypropan-1-one (DPEP) has not been established. The aim of the present study was to investigate the anticancer potential of DPEP and explore its possible mechanism of action.

## Material and Methods

### Material

**Figure 1:**
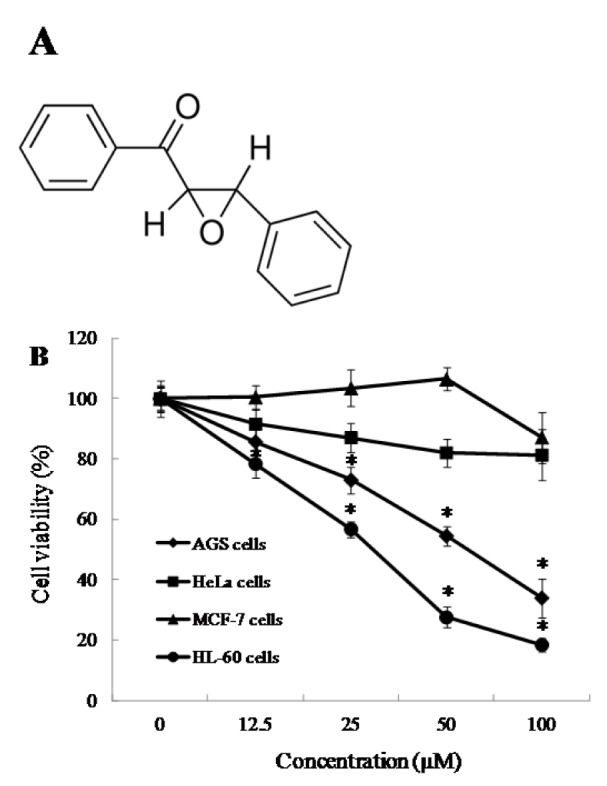
(A) Chemical structure of DPEP; (B) DPEP and its effect on various cancer cells viability. Cells in wells of 96-well plates were incubated with the various concentration of DPEP for 24 h. Cell viability was determined by a MTT assay. Each value indicates that the mean ± standard error from three independent experiments. *p < 0.05 indicate significant differences from control (without sample).

DPEP (Figure 1A(Fig. 1)), 3-(4,5-dimethylthiazol-2-yl)-2,5-diphenyltetrazolium bromide (MTT), RNase A, propidium iodide (PI), 2' 7'-dichlorodihydrofluorescein diacetate (DCFH_2_-DA), dimethyl sulfoxide (DMSO), N-acetylcysteine (NAC), phosphate buffered saline (PBS), RIPA buffer and Hoechst 33342 were purchased from Sigma-Aldrich (St. Louis, MO, USA). RPMI-1640 medium, Dulbecco's Modified Eagle's Medium (DMEM), fetal bovine serum (FBS), penicillin-streptomycin, and trypsin-EDTA were purchased from Gibco/BRL (Burlington, ON, Canada). Antibodies against Bax, Bcl-xL, cleaved capspase-3, PARP, and β-actin were obtained from Cell Signaling Technology (Bedford, MA, USA). All other chemicals and reagents used in these investigations were of analytical grade. 

### Cell culture 

HL-60 (a human promyelocytic leukemia cell line), AGS (a human gastric adenocarcinoma cell line), and MCF-7 (a human breast cancer cell line) cells were grown in RPMI-1640 medium, and HeLa (a human cervical carcinoma) cells were grown in DMEM supplemented with 10 % (v/v) heat-inactivated FBS, penicillin (100 U/mL), and streptomycin (100 μg/mL). Cultures were maintained at 37 °C in a 5 % CO_2_ incubator.

### Cell growth inhibition assay 

The cytotoxicity of DPEP against the tumor cells was assessed by a colorimetric MTT assay. Suspensions of HL-60 cells (2 × 10^5^ cells/mL) were seeded in multi-well plates and incubated with various concentrations of DPEP for up to 24 h prior to MTT treatment. Attached cells (AGS, MCF-7, and HeLa cells) were seeded in 96-well plates at a density of 1 × 10^5 ^cells/mL and incubated for 16 h. Following incubation, the cells were treated with various concentrations (12.5, 25, 50, and 100 μM) of the DPEP and subsequently incubated for an additional 24 h at 37 °C. MTT stock solution (50 μL; 2 mg/mL in PBS) was added to each well to achieve a total reaction volume of 250 μL. After 4 h of incubation, the plates were centrifuged for 10 min at 2,000 rpm, and the supernatants were aspirated. The formazan crystals in each well were dissolved in DMSO. The amount of purple formazan was assessed by measuring the absorbance at 540 nm. 

### Nuclear staining with Hoechst 33342 

HL-60 cells were seeded into 24-well plates at a density of 2 × 10^5^ cells/mL. The cells were then treated with various concentrations (20, 30, and 40 μM) of DPEP and incubated for an additional 12 h. Hoechst 33342 was added to the culture medium at a final concentration of 10 µg/mL, and the plates were incubated for an additional 10 min at 37 °C. The stained cells were then observed under a fluorescence microscope equipped with a CoolSNAP-Pro color digital camera to determine the degree of nuclear condensation. 

### Cell cycle analysis 

The HL-60 cells were seeded into 6-well plates at a density of 2 × 10^5 ^cells/mL. The cells were then treated with various concentration of DPEP and incubated for 12 h. The cells were harvested at the indicated times and fixed in 1 mL of 70 % ethanol for 30 min at 4 °C. The cells were washed twice with PBS and incubated in darkness with 1 ml of PBS containing 100 μg PI and 100 μg RNase A for 30 min at 37 °C. Flow cytometric analysis was conducted with an FACS Calibur flow cytometer (Becton Dickinson, San Jose, CA, USA). The effect of the extracts on the cell cycle was determined by changes in the cell distribution at each cell cycle phase, and assessed by histograms generated by the Quest and Mod-Fit computer programs. 

### ROS generation detection 

Intracellular ROS generation was measured by the DCFH_2_-DA method. HL-60 cells were seeded in 6-well plates at a density of 2 × 10^5 ^cells/mL. Cells were pretreated with NAC (1 mM) for 1 h followed by 40 μM DPEP for 2 h. Cells were washed with PBS twice and then incubated with 10 μM DCFH_2_-DA for 30 min at 37 °C. The labeled cells were then washed in PBS, and the fluorescence was analyzed using a flow cytometer. 

### Western blot analysis 

Cells (2 × 10^5 ^cells/mL) were treated with 40 μM DPEP in the absence or presence of NAC for difference times or 8 h. Cells were collected and washed twice in ice-cold PBS, then lysed in RIPA buffer on ice for 1 h. Cell lysates were collected via centrifugation, and the protein concentrations were determined using a Bio-Rad protein assay kit (Hercules, CA, USA). The lysates containing 20 μg of protein were separated on a 4-12 % Bis-Tris NuPAGE gel (Invetrogen, Carlsbad, CA, USA) and transferred using the iBlot transfer device (Invitrogen) program 3 for 7 minutes. The membrane was blocked with 5 % skimmed milk and then incubated with primary antibodies for Bax, Bcl-xL, cleaved caspase-3, PARP, and β-actin separately overnight at 4 °C. The membranes were washed with TTBS and incubated for 1 h at room temperature with the secondary antibodies. Signals were developed using an ECL Western blotting detection kit and exposed to X-ray films.

### Statistical analysis

All data are expressed as means ± SD significant differences between the groups were determined using the unpaired Student's *t*-test. A value of ^*^p < 0.05 was considered to be statistically significant. 

## Result

### Inhibitory effect of DPEP on the growth of human cancer cell lines

In this study, we evaluated the growth inhibitory activity of DPEP in human cancer cell lines, including HL-60, AGS, MCF-7, and HeLa cells. DPEP significantly suppressed the viability of HL60 and AGS cells, with inhibitory activity of 81.5 % and 66.0 %, respectively. However, DPEP was less effective in MCF-7 (12.9 %) and HeLa (18.7 %) cells at the highest dosage (100 μM) assessed (Figure 1B(Fig. 1)). The IC_50_ values of DPEP in HL-60 and AGS cells were 32.8 and 53.4 μM, respectively. In addition, DPEP exhibited no cytotoxicity in normal cell lines (Vero; monkey kidney cells) at the tested concentrations (data not shown). Thus, HL-60 cells were selected for use in further experiments.

### DPEP-induced apoptosis in HL-60 cells

**Figure 2:**
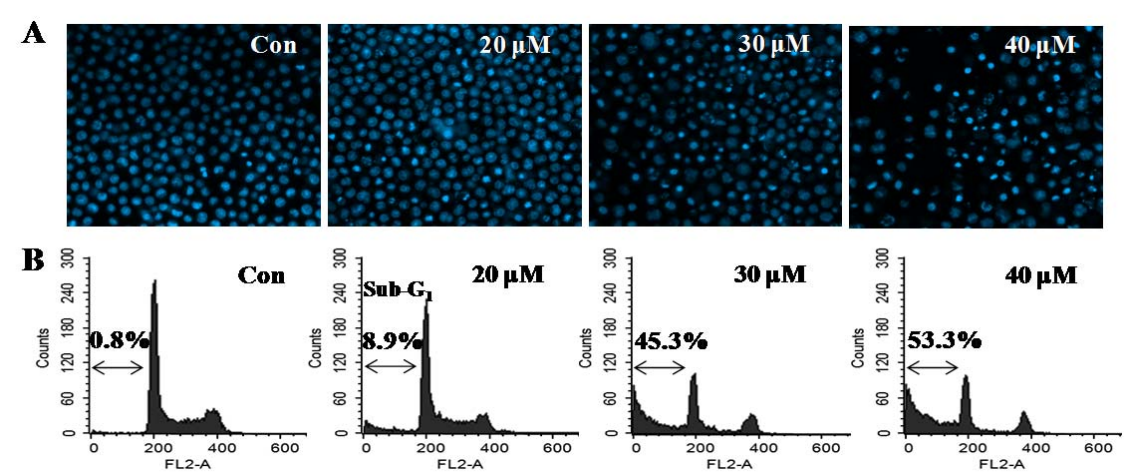
Induction of apoptosis by the DPEP treatment of HL-60 cells. HL-60 cells were seeded at 2 × 10^5^ cells/mL and treated with different DPEP concentrations for 12 h. (A) Apoptotic bodies were stained with Hoechst 33342 solution and observed under a fluorescent microscope using blue filter. (B) The cells were stained with PI and analyzed via flow cytometry. The experiment was repeated three independent times.

In order to determine whether the inhibitory effect of DPEP on cell viability was due to apoptosis, the HL-60 cells were treated with DPEP for 12 h, and Hoechst 33342 staining was performed. The controls, without DPEP exposure, exhibited no DNA damage (Figure 2A(Fig. 2)). However, obvious cell damage was observed in the DPEP-exposed cells. Cells treated with varying concentrations of DPEP (20, 30, and 40 μM) exhibited a dramatic increase in the number of apoptotic bodies (Figure 2A(Fig. 2)). Additionally, we measured the number of cells with sub-G1 DNA content, which is hypothesized to be apoptotic DNA, using flow cytometry. As shown in Figure 2B(Fig. 2), DPEP exposure increased the apoptotic portion of sub-G_1_ peaks in a dose-dependent manner (8.9 %, 45.3 %, and 53.3 % at concentrations of 20, 30, and 40 μM, respectively). Thus, apoptosis is correlated with the inhibition of cell growth, suggesting that HL-60 cells may undergo apoptosis after DPEP treatment.

### Effect of ROS on DPEP-induced apoptosis in HL-60 cells

**Figure 3:**
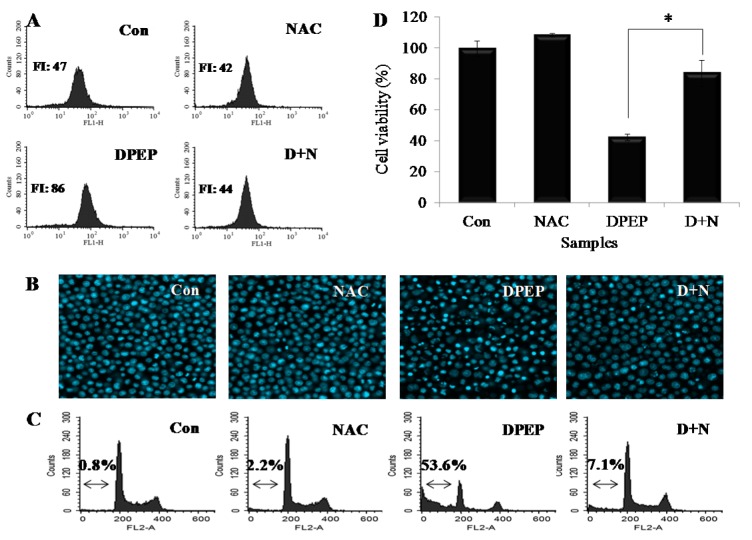
NAC pretreatment protected against DPEP-induced apoptosis via the suppression of ROS generation. HL-60 cells were pretreated with 1 mM NAC for 1 h prior to 2 h (A), 12 h (B, C), or 24 h (D) of 40 μM DPEP exposure. (A) The cells were labeled with 10 μM DCFH2-DA for 30 min at 37 °C, and subjected to subsequent FACS analyses for intracellular ROS accumulation. (B) Apoptotic bodies were stained with Hoechst 33342 solution and observed under a fluorescent microscope using a blue filter. (C) The cells were stained with PI and analyzed via flow cytometry. (D) Cell viability was measured via MTT assay. Each value indicates the mean ± standard error from three independent experiments. Difference with *p < 0.05 is considered statistically significant.

We used the fluorescent probe DCFH-DA to measure the generation of intracellular ROS in DPEP-treated HL-60 cells. As shown in Figure 3A(Fig. 3), DPEP significantly increased ROS production, as indicated by a rightward shift in fluorescence during the flow cytometric analysis. Moreover, pretreatment with the antioxidant NAC significantly reduced intracellular ROS levels compared to cells treated with DPEP alone. The inhibition of ROS generation by NAC also markedly prevented DPEP-induced apoptotic bodies (Figure 3B(Fig. 3)) and increased the percentage of sub-G1 cells (Figure 3C(Fig. 3)). NAC also inhibited DPEP-induced reductions in cell viability in HL-60 cells (Figure 3D(Fig. 3)). Together, these results suggest that intracellular ROS play an essential role in cell death by DPEP-induced apoptosis in HL-60 cells.

### Effect of DPEP and NAC on the expression of apoptosis-related protein

**Figure 4:**
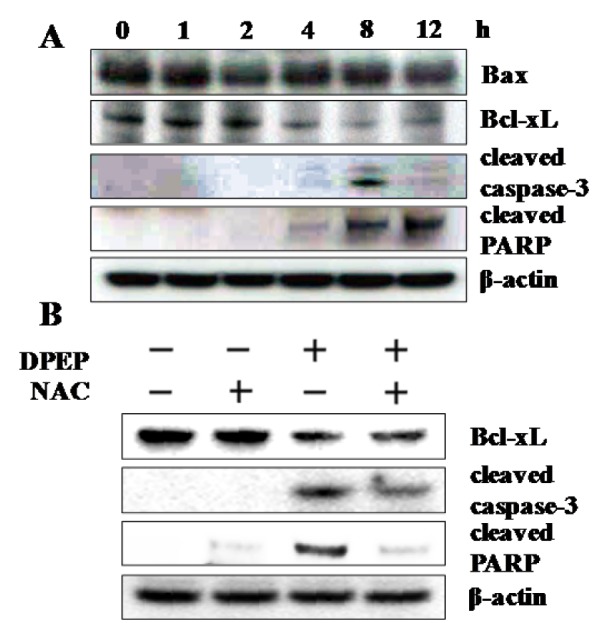
(A) Effect of DPEP on apoptosis-related protein in HL-60 cells. The cells were exposed to 40 μM DPEP for 1-12 h. (B) Effect of NAC on down-regulation of Bcl-xL and caspase-3, and PARP cleavage activation by DPEP. The cells were pretreated with 1 mM NAC and then treated for 8 h with 40 μM DPEP. Whole cell lysates were subjected to Western blot analysis of anti-Bax, -Bcl-xL, -cleaved-caspase -3, and -PARP monoclonal antibodies. β-Actin was used as internal control. The experiment was repeated three independent times.

Bcl-2 family proteins play a key role in the regulation of apoptosis by promoting (Bax and Bak) or inhibiting (Bcl-xL and Bcl-2) cell death. We therefore examined whether Bcl-xL played a role in DPEP-induced apoptosis. Western blot analysis revealed that DPEP treatment suppresses the expression of the anti-apoptotic Bcl-xL protein but did not affect the expression of the pro-apoptotic Bax protein. Further experiments revealed that DPEP induced cleavage of caspase-3 and PARP (Figure 4A(Fig. 4)). The results in Figure 3(Fig. 3) and 4A(Fig. 4) clearly show that both Bcl-xL and ROS are essential for DPEP-induced apoptosis, although the causal relationship between ROS and Bcl-xL is unclear. To further evaluate the significance of ROS generation in DPEP-mediated downregulation of Bcl-xL, HL-60 cells were pretreated with NAC for 1 h, followed by exposure to DPEP for 8 h. As shown in Figure 4B(Fig. 4), pretreatment with NAC attenuated caspase-3 and PARP cleavage and the reduction of Bcl-xL levels.

## Discussion

In this study, we found that DPEP decreased HL-60 cell viability in a dose-dependent manner via apoptosis. Moreover, we showed for the first time that DPEP-induced apoptosis of HL-60 cells occurs through ROS-mediated downregulation of Bcl-xL, suggesting that ROS act as upstream signaling molecules for the initiation of cell death.

Many chemotherapeutic agents are reported to exert their anti-cancer effects by inducing the apoptosis of cancer cells (Kamesaki, 1998[11]). Apoptosis is characterized by distinct morphological changes, including membrane blebbing, cytoplasmic shrinkage, dissipation of mitochondrial membrane potential, nuclear condensation, and DNA fragmentation (Hengartner, 2000[10]). In the present study, we showed that DPEP induced typical morphological characteristics of apoptosis, including nuclear condensation and apoptotic body formation in HL-60 cells. Additionally, DPEP-induced apoptosis of HL-60 cells was confirmed by the observation of sub-G_1_ DNA accumulation. These experiments indicated that DPEP induces cytotoxicity in HL-60 cells through a mechanism involving apoptosis. 

We next identified the cellular mechanism by which DPEP induced apoptosis in HL-60 cells. Bcl-2 family proteins play an important role in the regulation of apoptosis in mammalian cells (Adams and Cory, 1998[2]). Apoptosis is regulated by a delicate balance between homo- and hetero-dimerization of pro- and anti-apoptotic proteins. When the ratio of pro-apoptotic proteins exceeds anti-apoptotic proteins, pores form in the outer mitochondrial membrane, liberating apoptogenic mitochondrial proteins to activate caspases, thereby inducing apoptosis (Adams and Cory, 1998[2]). Bcl-xL, an anti-apoptotic protein, has been reported to play an important role in tumorigenesis and tumor progression. Bcl-xL may inhibit apoptosis by maintaining the permeabilization status or stabilizing the outer mitochondrial membrane (Boise et al., 1993[4]). Thus, we next sought to evaluate the effect of DPEP on Bcl-xL expression. Our results indicate that DPEP reduces Bcl-xL expression, without affecting Bax. In addition, DPEP-induced the activation of caspase-3 and cleavage of PARP. Caspase-3 is one of the key mediators of apoptosis, because it is either partially or totally responsible for the proteolytic cleavage of many key proteins, including PARP (Fernandes-Alnemri et al., 1994[9]). Cleavage of PARP leads to a number of cellular processes involving DNA repair and programmed cell death (Wang, 2001[23]). Hence, these data suggest that DPEP-induced apoptosis is mediated by the Bcl-xL pathway.

ROS generation plays a key role in apoptosis induced by various anticancer agents (Alexandre et al., 2007[3]; Sexton et al., 2006[19]). Previous reports indicate that the chalcone flavokawain B induced apoptosis through ROS induction in human colon cancer cells (Kuo et al., 2010[14]). Moreover, Winter et al. (2010[24]) reported that chalcone derivatives induced apoptosis via oxidative stress in leukemia cells. Our findings also demonstrated that DPEP treatment increased ROS levels in HL-60 cells, and that antioxidant pretreatment prevented ROS generation and apoptosis induced by DPEP. Many researchers have shown that natural and synthesized compounds induce apoptosis through the suppression of Bcl-xL via ROS production (Decaudin et al., 1997[8]; Li et al., 2004[15]; Woo et al., 2003[25]). Reports have also demonstrated that Bcl-xL-mediated caspase-3 activation is responsible for ROS-induced apoptosis in HL-60 cells (Kim et al., 2010[12]). Therefore, in order to better understand DPEP-induced ROS generation and Bcl-xL regulation, HL-60 cells were treated with DPEP in the presence or absence of NAC. DPEP-induced ROS generation downregulated Bcl-xL and caspase-3 expression and PARP cleavage. Quenching of ROS with NAC abolished DPEP-mediated Bcl-xL degradation and caspase-3 and PARP activation. Collectively, our findings support the notion that DPEP induces ROS generation, leading to the downregulation of the Bcl-xL pathway, triggering apoptosis of HL-60 cells. 

In conclusion, the chalcone derivative DPEP exhibited potential anti-cancer activity in human leukemia HL-60 cells through the induction of apoptosis. Moreover, we demonstrated that DPEP induced apoptosis via increased intracellular ROS, leading to inhibition of the Bcl-xL pathway. These findings may provide a deeper understanding of the mechanisms underlying the anti-cancer activity of DPEP.

## Acknowledgement

This research was supported by the project fund (C34290) to D. Kim from Korea Basic Science Institute. 

## Conflict of interest

The authors declare that they have no conflict of interest.

## Notes

Eun-Yi Ko and Seung-Hong Lee contributed equally to this work.

Daekyung Kim and Kil-Nam Kim (Jeju Center, Korea Basic Science Institute (KBSI), Jeju 690-140, Republic of Korea; Tel: +82-64-800-4933, E-mail: knkim@kbsi.re.kr) contributed equally as corresponding authors.
